# Warthin-Like Variant of Papillary Thyroid Carcinoma: An Uncommon Variant

**DOI:** 10.7759/cureus.12350

**Published:** 2020-12-28

**Authors:** Tarun Kumar, Jitendra S Nigam, Prerna Tewari, Chandan K Jha

**Affiliations:** 1 Pathology, All India Institute of Medical Sciences, Patna, IND; 2 Pathology/Lab Medicine, All India Institute of Medical Sciences, Patna, IND; 3 Endocrine Surgery, All India Institute of Medical Sciences, Patna, IND

**Keywords:** warthin tumor, salivary gland, thyroid, cytology

## Abstract

Papillary thyroid carcinoma (PTC) is one of the most common thyroid malignancy with various histologic variants. Acknowledging the correct histological variant of PTC helps to know about the tumor's nature and prognosis. The Warthin-like variant of papillary thyroid (WLPTC), a newly described histologic variant of PTC, is relatively uncommon. A 16-year-old female presented with complaints of painful thyroid swelling for two years. Fine needle aspiration cytology (FNAC) from the lobes showed lymphocytic thyroiditis features with Hurthle cell change. Sections from the left lobe revealed a diagnosis of a Warthin‑like variant of PTC without nodal metastasis. WLPTC is a rare variant having a favorable outcome due to the absence of lymph node metastasis, extra-thyroidal extension, and a low recurrence rate. The correct cytological and histomorphological features are of utmost importance to render the diagnosis of WLPTC for better management.

## Introduction

Papillary thyroid carcinoma (PTC) is the most common malignant tumor of the thyroid [1]. There are approximately 15 histologic variants of PTC having variable biological behavior and prognosis [1]. The Warthin-like variant of papillary thyroid (WLPTC) is an uncommon tumor characterized by papillae lined by large oncocytic cells with cores having dense lymphoplasmacytic infiltrate [1,2]. These tumors have a resemblance to the Warthin tumor of salivary gland origin [1]. The prognosis of WLPTC is the same or less aggressive than that of classical PTC [2-4]. We are presenting a case of WLPTC due to its rarity.

## Case presentation

**Figure 1:**
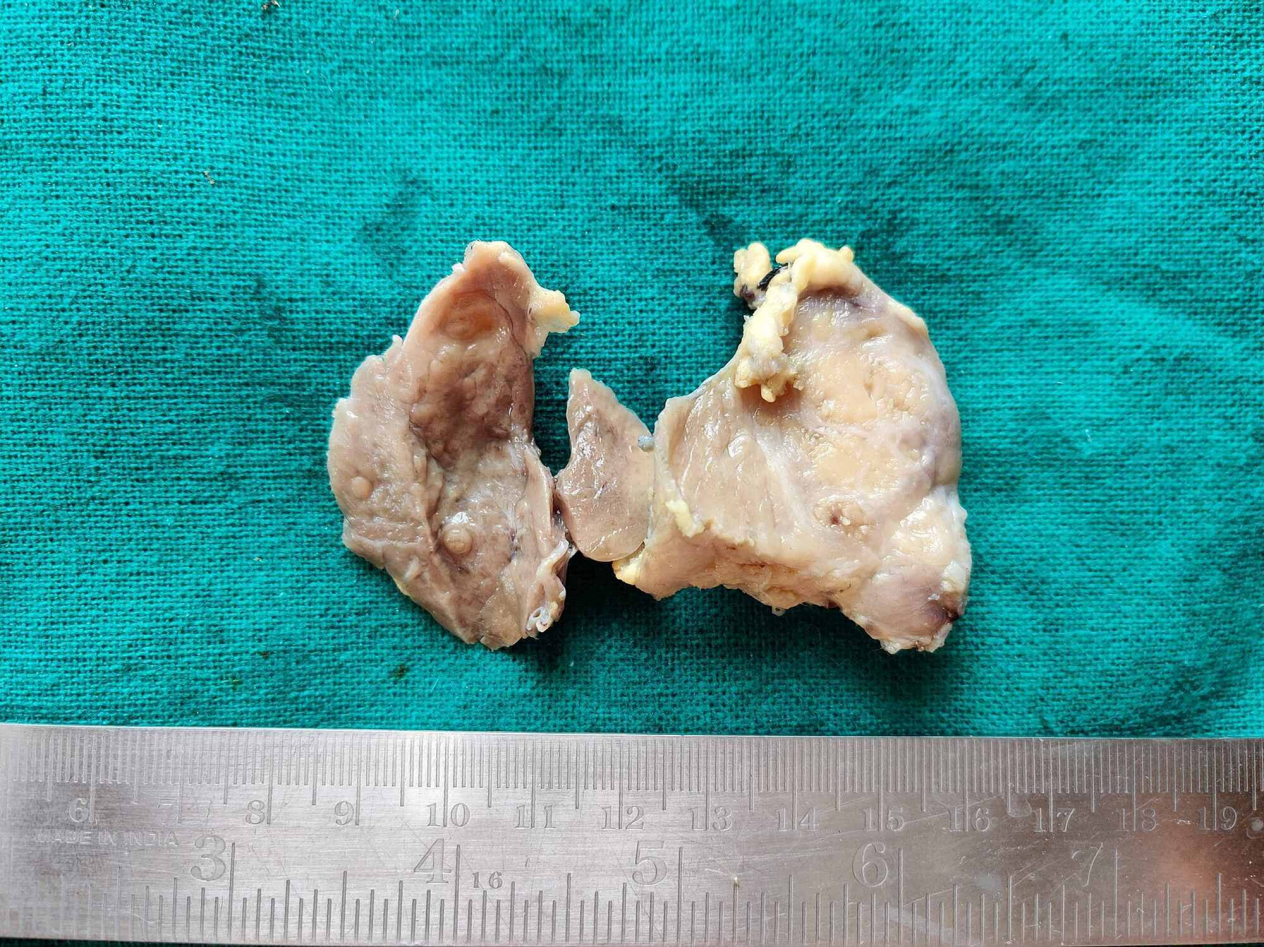
Gross: The cut surface of thyroid specimen Left lobe: Solid, poorly circumscribed grayish-white lesion. Right lobe: Gray-brown homogenous areas with two small nodules. Isthmus: Grayish brown homogenous area.

A 16-year-old female presented with complaints of painful thyroid swelling for two years. She did not give any previous medical, surgical, or family history of thyroid lesion. Physical examination revealed diffuse, firm to hard enlargement of the thyroid (left>right). No neck nodes were palpable. Thyroid function tests were FT3 -- 4.36 pmol/L (3.5-6.5 pmol/L), FT4 -- 16.48 pmol/L (11.5-22.7 pmol/L) and TSH -- 2.07 IU/mL (0.33-5.5 IU/mL). Ultrasonography (USG) of the neck showed diffuse enlargement of the thyroid gland with extensive vascularity suggestive of multinodular goiter. Fine needle aspiration cytology (FNAC) from both lobes showed lymphocytic thyroiditis features with Hurthle cell change (Figure 1).

**Figure 2:**
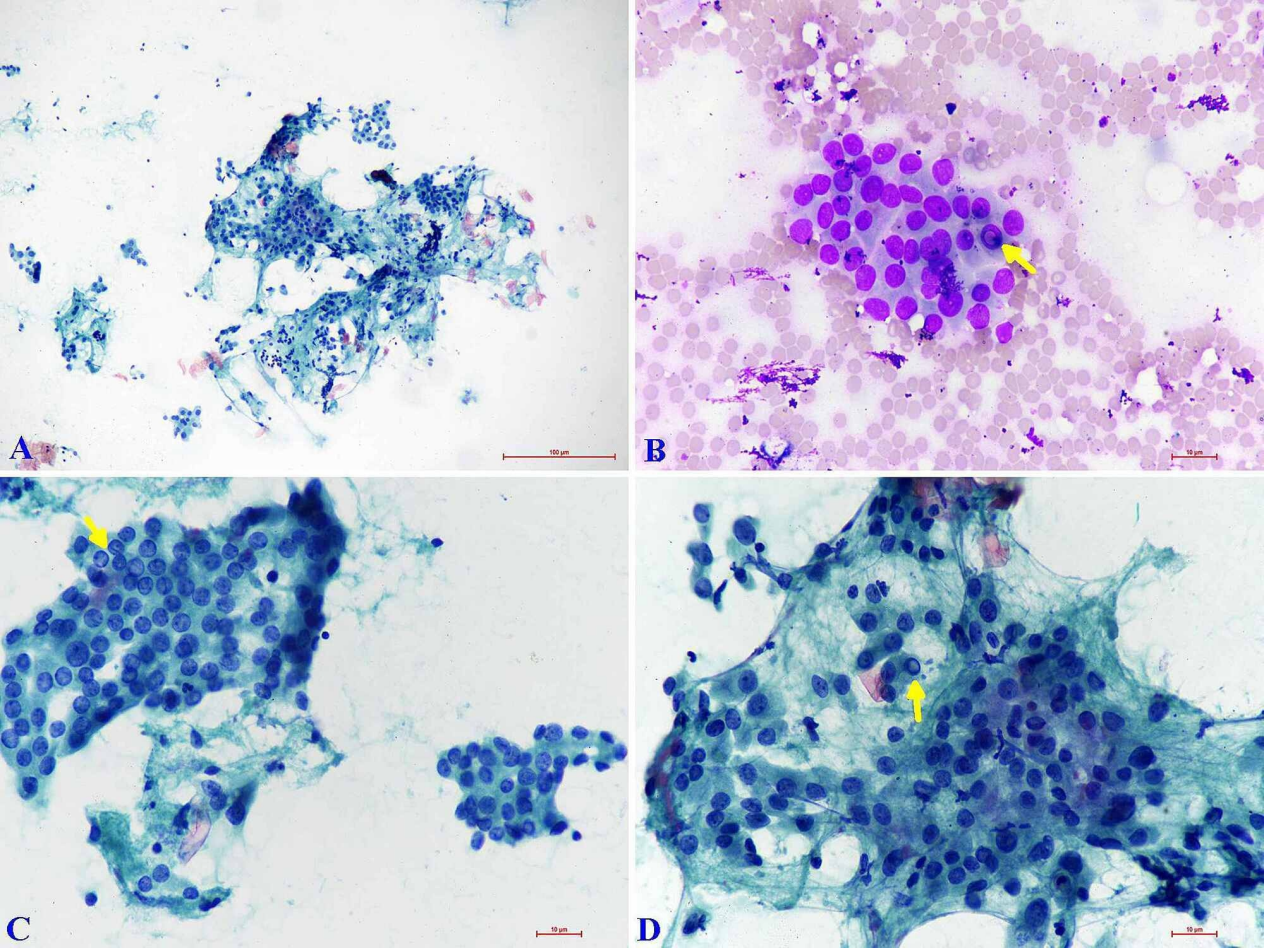
Cytology (A) Cellular smears showing monolayer sheets of follicular cells with lymphocyte impingement. (Papanicolaou stain: x40) (B) Follicular cells with Hurthle cell change and occasional intranuclear inclusion (arrow). (Giemsa: x400) (C & D) Follicular cells with mild nucleomegaly having vesicular chromatin, moderate cytoplasm, and an occasional intranuclear inclusion (arrow). (Papanicolaou stain: x400)

The patient underwent total thyroidectomy without neck lymph node clearance. Grossly, total thyroidectomy specimen with left lobe, the right lobe, and isthmus measured 4x2.5x3 cm, 4x2.5x1.2 cm, and 1.5x1.0x0.5 cm, respectively. The cut surface of the left lobe showed poorly circumscribed, grayish-white solid areas. The right lobe cut surface showed homogenous, gray-brown areas with two small nodules, each measuring 0.4x0.4x0.2 cm. The cut surface of the isthmus showed grayish-brown (Figure 2).

**Figure 3:**
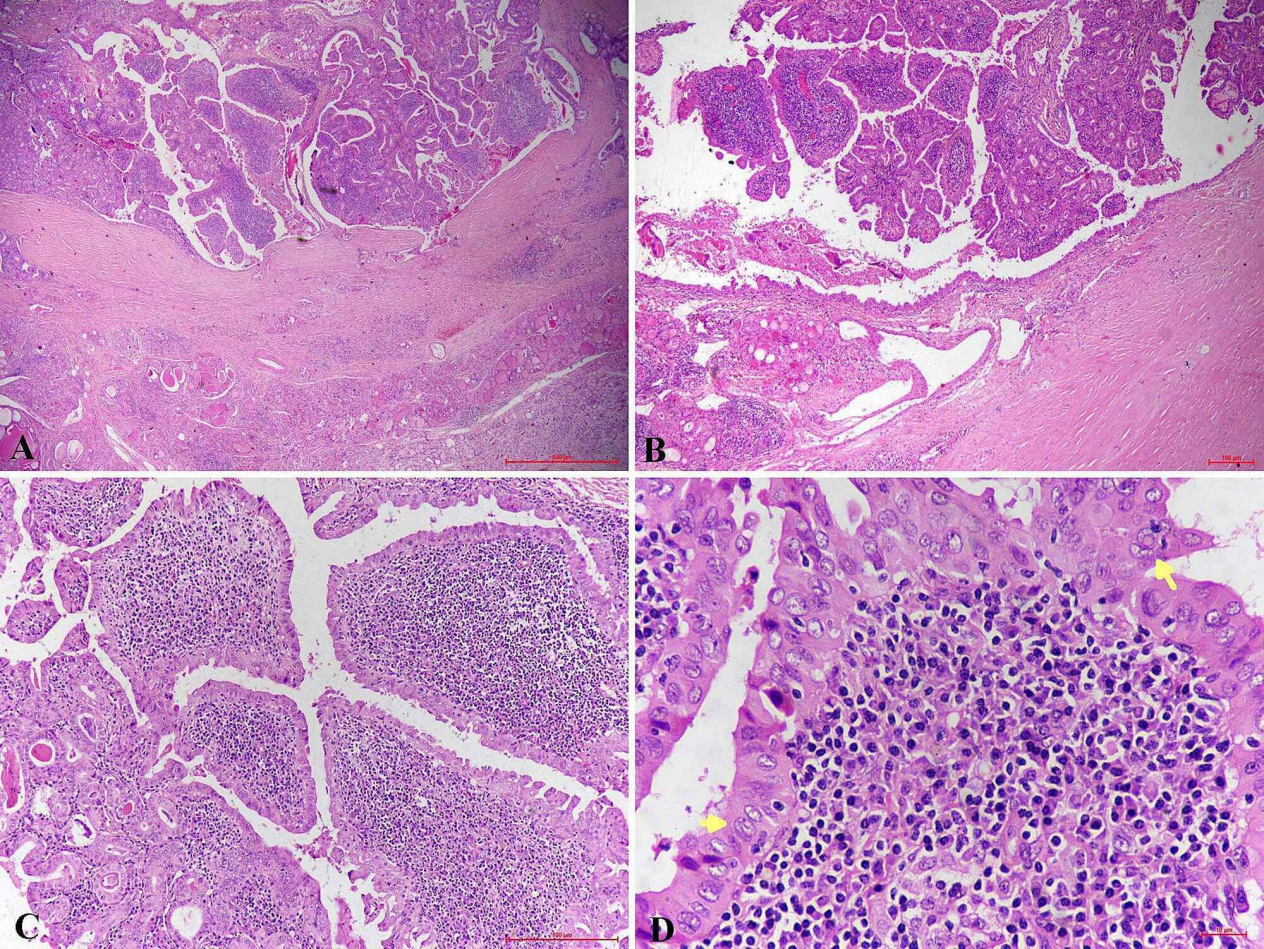
Histology (A & B) Poorly circumscribed tumor mass showing numerous papillae. The peritumoral area shows lymphocytic thyroiditis. (C) Papillae lined by Hurthle cells with stalk showing dense lymphoplasmacytic infiltrate. (D) Nuclear features of PTC like Intranuclear pseudo inclusion (arrow) and nuclear groove (arrowhead). PTC: papillary thyroid carcinoma

Sections from the left lobe showed a tumor composed of papillae lined by Hurthle cells having an abundant amount of granular eosinophilic cytoplasm and nuclear clearing and grooving with intranuclear cytoplasmic inclusions. The stalk of papillae showed dense lymphoplasmacytic infiltrate (Figure 3).

Sections from the right lobe and Isthmus showed features of lymphocytic thyroiditis. Based on histomorphology, a diagnosis of a Warthin-like variant of PTC of the left lobe was made. Post-operative six months follow-up was uneventful.

## Discussion

In 1995, Apel et al. first described this variant as "papillary Hurthle cell carcinoma of the thyroid with lymphocytic stroma Warthin-like tumor of the thyroid" [2]. Later it was called WLPTC due to its histological resemblance to the Warthin tumor of the salivary gland [2]. The prevalence of WLPTC ranges from 0.2% to 1.9%, which may be due to misclassification into classical, oncocytic, or Tall cell variant [5-7]. The age of onset ranges from the second to sixth decade (mean: 44.9 years) with a female preponderance [5]. The tumor size varies in range from 3 mm to 22 mm with a mean of 8.9 mm [5]. FNAC features were first described by Youseff et al. in 1997, which includes papillary clusters or monolayered sheets of Hurthle cells with nuclear features of PTC and dense lymphoid cell population in the background [8]. Thus, it shows characteristics of both PTC and Hashimoto thyroiditis [8]. The presence of both features is of utmost essential for cytological diagnosis [8]. In the present case, the patient was 16 years old female harboring a tumor size of 40 mm, showing a cellular smear composed of monolayer sheets of Hurthle cells lacking PTC's nuclear features with the presence of lymphocytic infiltrate. Histologically, WLPTC is characterized by papillae lined by large oncocytic cells with cores having dense lymphoplasmacytic infiltrate [1,2]. Various studies hypothesized that lymphocytic infiltration is due to the autoimmune mechanism by the presence of RET/PTC fusion gene of PTC [9,10]. The differential diagnosis of WLPTC includes Hashimoto thyroiditis, Hurthle cell neoplasm, classical PTC arising in thyroiditis background, tall cell variant, and the oxyphilic variant of PTC [11]. Acknowledging the correct histomorphology has of utmost importance as some of the mimickers have more aggressive and unfavorable outcomes than WLPTC [12]. WLPTC has a favorable outcome due to the absence of lymph node metastasis, extra-thyroidal extension, and a low recurrence rate [13]. Tall cell variants have papillary architecture lined by oncocytes with a height of more than two to three times the width [14]. Oxyphilic variants have papillary architecture lined by Hurthle cell, showing PTC nuclear features with nuclear atypia and hyperchromasia. There is an absence of dense lymphoplasmacytic infiltrate [15]. In the present case, PTC's nuclear features, dense lymphoplasmacytic infiltrate in the papillary core, help to differentiate from Hashimoto thyroiditis, Hurthle cell neoplasm, classical, tall cell, and the oncocytic variant of PTC. As histomorphology is quite characteristics, the role of immunohistochemistry is minimal for a definite diagnosis [3]. BRAF V600E mutation has been implicated in the pathogenesis of classical PTC, while 75% of WLPTC variant harbor BRAF V600E mutation [16-18]. Depending upon the tumor stage at the time of diagnosis, family history, and neck irradiation history, Total thyroidectomy with/without lymph node dissection is the treatment modality [3]. Post-operative, radioiodine ablation therapy and regular follow-up are employed in high-risk patients having residual thyroid tissue and capsular invasion [3]. The present case was reported because of its rarity as well as to emphasize the problems in the preoperative cytological diagnosis of this rare tumor. Although the sensitivity and specificity of FNAC in the diagnosis of PTC are high, some rare variants like WLPTC may be missed on FNAC [8,19].

## Conclusions

WLPTC is an uncommon variant of PTC regarded as a distinct entity with an excellent prognosis. Hence, the pathologist must identify the cytological and histomorphological features to render the correct diagnosis for better management.
